# Thermo-sensitive hydrogel combined with SHH expressed RMSCs for rat spinal cord regeneration

**DOI:** 10.3389/fbioe.2022.1001396

**Published:** 2022-10-21

**Authors:** Jun Gu, Biao Gao, Hajra Zafar, Bo Chu, Xiaojun Feng, Yinjie Ni, Lin Xu, Rui Bao

**Affiliations:** ^1^ School of Medicine, Yangzhou University, Yangzhou, China; ^2^ Department of Orthopedics, Xishan People’s Hospital, Wuxi, China; ^3^ Wuxi Xishan District Ehu Town Health Center, Wuxi, China; ^4^ School of Pharmacy, Shanghai Jiao Tong University, Shanghai, China; ^5^ Department of Pharmaceutics, School of Pharmacy, Jiangsu University, Zhenjiang, China

**Keywords:** spinal cord injury, thermo-sensitive hydrogel, Shh, rat bone marrow mesenchymal stem cells, nerve regeneration

## Abstract

**Purpose:** Spinal cord injury (SCI) has a damaging impact on patients, amid being a worldwide problem with no effective treatment. Herein, we reported a method for functional therapy of SCI in rats, wherein we combined thermo-sensitive hydrogel with Sonic Hedgehog (SHH) expressed in rat bone-marrow derived mesenchymal stem cells (RMSCs).

**Methods:** Bone marrow-derived mesenchymal stem cells (BMSCs) were isolated from Sprague-Dawley (SD) female rats. The SHH was optimized and transferred into RMSCs *via* cationic liposomes, while thermo-sensitive hydrogel was reformed with hyaluronate (HA) and Pluronic F127. Then, a rat model with SCI was established accordingly by male SD rats and randomized into sham, model, RMSCs with hydrogel and SHH-RMSCs with hydrogel. The evaluation of SCI repair based on Basso, Beattie Bresnahanlocomotor rating scale (BBB scale) and inclined plate score. Immunofluorescence, immunohistochemistry and hematoxylin-eosin were utilized to explore the expression of protein (GFAP, GAP43, NF200 and MBP) and histopathology.

**Results:** It was demonstrated that transfection of SHH with cationic liposomes exhibited more effect in RMSCs than lipofectamine 2000. As shown in SEM, 3.5% HA-F127 demonstrated porous structure. In the MTT and dead/live assay, 3.5% HA-F127 showed good biocompatibility for RMSCs. Both RMSCs and SHH-RMSCs groups could significantly promote BBB and inclined plate scores (*p* < 0.01) compared with the model. Furthermore, the SHH-RMSC group was significantly improved than RMSC with the expression of related proteins, where NF200, MBP, and GAP43 were principally enhanced with the GFAP expression being virtually down-regulated.

**Conclusion:** All in all, the results suggested that transplantation of RMSCs with SHH could improve the function of SCI and promote nerve regeneration.

## 1 Introduction

The pathological process of traumatic injuries such as spinal cord injury (SCI) is complicated coupled with high disability and mortality. SCI often leads to sensory and motor deficits, as well as acutely causing bradyarrhythmia, vasodilation and autonomic dysreflexia, ectopic beats, hypotension, and neurogenic shock (Hagen, 2015; Taweel and Seyam, 2015; Villanova Junior et al., 2020). Additionally, SCI could be continuously developed in different time stages, leading to expansion of the initial injured area (Wills and Ninness, 2012) and harsh treatment environment. After injury to the central nervous system (CNS), the lost axons cannot be regenerated due to intrinsic limitations. The failure of axonal regeneration is due to the lack of neurotrophic support (Widenfalk et al., 2001), growth-inhibitory molecules (Kerstetter and Miller, 2012) and the absence of the immune function (Schwartz, 2000). Meanwhile, glial cells could produce growth-inhibitors which prevent axon regeneration, namely Nogo (Schnell and Schwab, 1990), MAG (Reindl and Waters, 2019), tenascin (Becker et al., 2000), and proteoglycans of chondroitin sulfate (Lemons et al., 1999).

Current treatment options for SCI include the use of high-dose sodium succinate methylprednisolone and early surgery, which aim to prevent further injury. Because the poor neurologic recovery affecting the patients daily lives, it is very worthwhile to find a more effective treatment method for spinal cord injury (Chhabra and Arora, 2012). Therefore, effective therapeutic modalities are essential to protect neurons from secondary injury and promote neurologic recovery. Cell transplantation has become a promising therapy for SCI. It has been widely recognized that cell transplantation could survive adequately for a longer period to facilitate the regeneration of axons sufficiently and appropriately (Neuhuber et al., 2005).

As mesenchymal stem cells, marrow stromal cells (MSCs) have multiple abilities to differentiate into triploids, such as bone (Beresford et al., 1992), cartilage (Lennon et al., 1995), fat (Beresford et al., 1992) and muscle (Wakitani et al., 1995). Bone marrow-derived mesenchymal stem cells (RMSCs) also have the ability to differentiate along three essential mesenchymal lineages: osteoblasts, adipocytes, and chondroblasts (Pittenger et al., 1999; Rubio et al., 2005). Importantly, it had been reported that MSCs have potential to differentiate into nerve cells. Related studies reported that injection of MSCs into the SCI rat could improve motor function, while accelerating recovery of the rat motor during spinal transplantation with moderate SCI in the basso, as evidenced by the BBB score system (Lin et al., 2018). And there have also been reports showing that wound healing is achieved effectively with the application of BMSCs (Han et al., 2005; Falanga et al., 2007).

In recent years, genetic engineering technology has become more and more prosperous and has been applied in the treatment of clinical diseases. At present, vectors of gene therapy are mainly allocated into two classes, namely non-viral and viral vectors. In particular, viral vectors have the advantages of high transfection and transformation rates, but clinical applications have been limited owing to their small capacity, difficult preparation, high immunogenicity, and other potential safety issues. Comparably, the non-viral vectors have the advantages of natural degradation, simple preparation, low toxicity and repeatable transfection, especially low immunogenicity (Zdanov et al., 2000). Common non-viral vectors include cationic liposomes, naked DNA and cationic polymers, among which cationic liposomes are more applicable. Liposomes are composed of a phospholipid structure similar to that of a biological membrane and other lipids. The liposome forms single-layer or multilayer microcapsules dispersed in water. Cationic liposomes usually composed of positively charged amphoteric compounds and neutral lipid with dispersive properties of nanoparticles. The basic structure of cationic liposome is that the positively charged group is connected to the hydrophobic group. Because of the positive charge, cationic liposome can combine the negative charged target gene to form a stable complex, which can transfer a variety of substances into various types of cells, such as animals, plants, microorganisms, etc.

Hydrogels can form polymer chains that cross physically or chemically the forming 3D-porous structure (3D)-porous structure (Liu et al., 2018). In view of their high biocompatibility, abundant aquatic content, and mechanical properties, hydrogels can act as an ideal scaffold candidate for tissue engineering and other important biological functions such as nerve cell adhesion, exchange of metabolic substances, and loading of bioactive compounds for SCI regeneration (Zhou et al., 2018; Zhang et al., 2021). We hypothesized that combination of hydrogels and other delivery systems like liposomes may enhance SCI treatment.

Sonic hedgehog (SHH) is an essential signal in the formation of the neuronal pattern, regulation of cell fate, axonal guidance, proliferation, survival and differentiation during the development of the central nervous system (CNS). Gli is the downstream signal molecule of SHH, which has been shown to promote the protective effect of SCI recovery, amid the potential to maintain biological activity at the injury site for the short term. Presently, available literature suggests that signal of SHH/Gli1 in activated astrocytes plays a crucial role in blood-SC barrier permeability and recovery of locomotor function after SCI (Yue et al., 2020). Furthermore, SHH chitosan microspheres (SHH/CS) embedded in fibrin scaffolds could provide protection and regeneration for complete transection of SCI in rats (Liu et al., 2018), thus achieving better therapeutic effects than without microsphere scaffold (Yang et al., 2020).

In this study, cationic liposomes were formed by the thin film dispersion method, while RMSCs were transfected with SHH gene that was delivered by cationic liposomes. The expression of SHH gene in RMSCs was optimized and combined with thermo-sensitive hydrogel scaffold, which may provide suitable scaffold materials for repairing SCI in rats, wherein the repair effect was investigated.

## 2 Materials and methods

### 2.1 Materials

Merck KGaA (Darmstadt, Germany) supplied 1,2-dioleoyl-3-trimethylammonium-propane (DOTAP) and 1, 2-dioleoyl-sn-glycero-3-phosphocholine (DOPC), while SinoPharm Chemical Reagent Co., Ltd. Provide chloroform and ethanol. Chromatographically grade methanol was supplied by Hanbon Sci. And Tech. (Jiangsu, China). Production of double distilled (DD) water was accomplished with a Millipore Water Purifying System (Millipore Corp., Bedford, MA, United States). Other analytically grade chemicals were obtained commercially. We bought MSCgo™ Rapid Osteogenic Differentiation Medium, fetal bovine serum (FBS), DMEM medium and D-hanks solution from Biological Industries (Israel). Anti-NF200 (#55453,1:200 dilution for Immunofluorescence), anti-GAP43 (#8945, 1:200 dilution for Immunofluorescence), anti-GFAP (#80788, 1:200 dilution for Immunofluorescence), anti-MBP (#78896, 1:200 dilution for Immunofluorescence), anti-CD44-APC-conjugate (#80813), mAb IgG2b Isotype Control (APC Conjugate) (#34828) and alexa Fluor 488 goat anti-rabbit IgG (#4412,1:500 dilution) were purchased from Cell Signaling Technology Co., Ltd. Anti-CD29-APC-conjugate (17-0291-82), anti-vimentin-APC-conjugate (MA5-28601), TGF-beta1, oil red O, HA and F127 were obtained from Sigma Co., Ltd. All the antibodies were obtained from Cell Signaling Technology, Inc. The DsDNA fluorimetry test kits were obtained by Jiaxing Yakangbo Medical Examination Co., Ltd.

### 2.2 Animals

The Center for Laboratory Animal Research at Jiangsu University (Zhenjiang, China) supplied the male SD rats (200–250 g, 14–16 weeks) for establishment of SCI model and the female SD rats (180–230 g, 6–8 weeks) for isolating RMSCs. We maintained the rats for 3 days at room temperature, 40%–70% relative humidity and 12/12 cycle of ligh/dark with free water and food access. The animal study was reviewed and approved by the board of institutional review at the Institute of Jiangsu University.

### 2.3 Isolation and purification of RMSCs

RMSCs were harvested from adult male SD rats (6–8 weeks). In brief, SD rats were applied for intraperitoneal anaesthesia (1% pentobarbital sodium, 50 mg/kg). And femurs and tibias were cut off aseptically, removed the skin and muscles. A hole was then created in the knee joint end of each bone with a 26-gauge needle, and marrow was flushed with D-hanks solution containing 2% FBS. The solution was centrifuged for 5 min at 800 rpm. The supernatant was aspirated gently, and pallet was resuspended in DMEM comprising streptomycin (100 μg/mL) penicillin (100 U/mL), and 10% FBS. Cells were seeded in tissue culture flask and cultured in incubator with 5% CO_2_, and 37°C. After 24 h, the non-adherent cells were discarded after medium refreshing. Then, the culture medium was refreshed every 3 days. After RMSCs had achieved 80% confluence, we digested and passaged the cells at a density of 2.0 × 10^5^ cells per ml for subculturing. The cells morphology and growth were observed under a microscope.

### 2.4 Characterization of RMSCs

#### 2.4.1 Alizarin Red staining of RMSCs

Seeding of RMSCs (4.5 × 10^4^ cells per mL) in a 6-well plate with MSCgo™ Rapid Osteogenic Differentiation Medium was accomplished before incubation under 5% CO_2_ at 37°C, while medium renewal was carried out once in 3 days. Later, we fixed the cells after 10 days with ethanol (95%) for 10 min, before addition of Alizarin Red staining solution and 30 min of incubation at 37°C. After twice washing, the stained cells were detected under optical microscope.

#### 2.4.2 Oil red O staining of RMSCs

Seeding of RMSCs (4.5 × 10^4^ cells per mL) in a 6-well plate was carried out for 10 days with DMEM, which contained 3-isobutyl-1-methyl-xanthine (IBMX, 0.5 mmol/L), insulin (5 μg/mL), and dexamethasone (1 µM) for inducing adipogenic differentiation, while the medium was renewed once in 3 days. The cells were then stained with oil red O for 15 min, rinsed with 85% propylene glycol for 3 min, before washing in DD water for 3 times and recording with optical microscope.

#### 2.4.3 Type II collagen-FITC and makers staining

Seeding of RMSCs (4.5 × 10^4^ cells per mL) in a 6-well plate was carried out for 2 weeks in DMEM medium that contained insulin (6.25 mmol/L), transforming growth factor β1 (TGF-β1, 10 μg/L), and 10% FBS (v/v), as well as subsequent cell inducement and assays with Type II collagen-FITC. Meanwhile.

#### 2.4.4 Flow cytometry analysis

Detection of cell surface markers (CD29 and CD44) and intracellular markers (vimentin) was performed by flow cytometry antibody staining as following procedure. RMSCs were incubated with APC-conjugated mAb, including anti-CD29, anti-CD44, anti-vimentin. Data were acquired and analyzed on a BD Accuri TM C6 Plus with FlowJo V10 software.

#### 2.4.5 Growth curve of RMSCs

RMSCs were digested into single-cell suspension and 5 min of centrifugation at 1000 rpm. The precipitated cells was resuspended and counted, while the adjusted cells (1 × 10^4^ cells per mL) were inoculated in a 24-well plate. Meanwhile, the cells were counted every day for a week in triplicate, before we plotted growth curve with abscissa representing culture time and ordinate denoting the average number of cells.

### 2.5 Cationic liposomal preparation and characterization

#### 2.5.1 Preparation of cationic liposome

The preparation of cationic liposomes was carried out using the thin film dispersion method (Zhong et al., 2007) (28). The brief method was described as follows: DOTAP (350 mg) and DOPC (370 mg) were dissolved in chloroform (10 ml) prior to 10 min of vortexing. Evaporation of solvent was performed at 50°C with a rotary evaporator to form a thin-film of DOTAP and DOPC on tube wall. Later, we dried the film for 1 h to remove residual solvent. We bruised the lipid film into powder before it was dissolved in DD water (4 ml) under vigorous stirring to form cationic liposomes.

#### 2.5.2 Cationic liposomal characterization

Analysis of particle size and zeta potential was carried out *via* laser light scattering Zetasizer (Brook-haven Instruments Corporation, Holtsville, NY, United States) at temperature of 25°C and angle of 90° by diluting with DD water (1:8, v/v) as previously described (Bao et al., 2020)(29). Morphological visualization of diluted cationic liposomes with DD water (1:100, v/v) was accomplished under transmission electron microscopy (TEM, JEM-2100, JEOL, Tokyo, Japan). Briefly, we added phosphotungstate solution (2%) to stain the cationic liposomes after placement on copper mesh. Later, the liposomes were observed by the TEM after they have been dried.

### 2.6 Transfer SHH gene into RMSCs *via* cationic liposome

#### 2.6.1 Cell culture and transfection

Plasmids carrying the SHH gene were constructed by Sangon Biotech, shanghai, China. The third generation RMSCs was transfected with SHH plasmids *in vitro*. The experiment was randomized into three groups, *viz.*, experimental (cationic liposome transfected SHH), control (cationic liposome) and positive control groups (Lipofectamine 2,000 transfected SHH). In experimental group, different concentrations of SHH plasmid were mixed with cationic liposome solution (without FBS) at room temperature (RT) and incubated for 20 min. This mixture was added to RMSCs and cultured in the incubator. After 4 h, we replaced the culture medium with fresh DMEM. Afterwards, cultivation of the cells was carried out for 8 days prior to testing.

#### 2.6.2 Optimal transfection efficiency of cationic liposomes

Different concentrations of SHH plasmids (20, 40, 80, and 160 ng) were mixed with cationic liposomes, while different RMSCs numbers (1 × 10^4^, 2 × 10^4^, 4 × 10^4^ and 8 × 10^4^ cells per well) were explored. The procedure was in accordance with the above method (Section 2.6.1). The SHH was detected by ELISA and Western blot (WB) to optimize the transfection condition, namely, the concentration of plasmids and the number of cells.

#### 2.6.3 Transfected RMSCs

RMSCs (transfected with an optimal ratio of SHH-liposome) were seeded in a 24 well plate (500 µl per well) at 2 × 10^4^ cells per mL density. Next, the cells were cultivated for 8 days, before detection in triplicate (every day) of the growth curve.

### 2.7 Establishment of temperature sensitive gel

#### 2.7.1 Preparation of temperature sensitive gel

Amounts of Pluronic F127 (0.6 g) and sodium hyaluronate (0.025, 0.05, 0.075, 0.1, 0.125, 0.15, 0.175, and 0.2 g, HA-F127 solution with mass fractions of 12% for F-127 and 0.5%, 1.0%, 1.5%, 2.0%, 2.5%, 3.0%, 3.5%, and 4.0% for hyaluronate) were weighed and mixed at 4°C. Next, the mixed HA-F127 solution was placed under a 37°C water bath to gelatinize, while the transaction time was recorded, respectively.

#### 2.7.2 Observing the morphology by SEM

The HA-F127 hydrogel was lyophilized by the freeze dryer. The lyophilized material was then placed on conductive tape and metal sprayed for 60 s to observe the morphology *via* a Philips XL-30E scanning electron microscope.

#### 2.7.3 Mechanical properties

For competition swelling, we placed the HA-F127 hydrogel in PBS (pH 7.4) for 24 h at room temperature. Using a universal material testing machine, we subjected the gel to a mechanical compression test at room temperature, wherein the compression speed was 2 mm/min. Each experimental group was repeated in triplicate and the average value was recorded.

#### 2.7.4 Swelling performance

The HA-F127 hydrogel was immersed in PBS (pH7.4) and placed in a 37°C thermostat air bath to completely expand. After 24 h, the samples were taken out, before we used filter paper to absorb water on the surface. Later, we accurately weighed the swelled hydrogels as W1, while the samples were lyophilized totally and weighed as W2. The calculation of the swelling ratio (SR) of the HA-F127 hydrogel was performed using formula (Villanova Junior et al., 2020), while the samples were measured in triplicate.

$$SR=\frac{W_{1}-W_{2}}{W_{2}}$$ (1)

#### 2.7.5 *In vitro* degradation test

The HA-F127 hydrogel was added to PBS (pH7.4) before swelling in a constant temperature shaking box at 37°C for 24 h. Afterwards, the hydrogel was taken out, wiped with filter paper to remove residual surface moisture, before weighing the hydrogel mass as W_a_. The hydrogel was placed in a 0.02% (w/v) sodium azide solution in PBS before shaking at 37°C, while the hydrogel was weighed and recorded as Wb at different times. The remaining mass (RM %) of the hydrogel degradation over time was calculated by the following formula (Taweel and Seyam, 2015). All samples were measured in triplicate before statistical comparison and analysis.

$$RM\;\%=\frac{W_{a}}{W_{b}}\times 100\%$$ (2)

#### 2.7.6 Release test

The HA-F127 hydrogel containing SHH plasmid was collected in a tube comprising PBS (2 ml, pH = 7.4) before placement in a shaker operating at constant temperature of 37°C. Aliquot (300 μl) of release solution was withdrawn at 1, 2, 3, 4, 5, 6, 7, and 8 days, prior to supplementation with equal amount of fresh PBS (pH = 7.4) in the tube. Ds-DNA kit was used to determine the concentration of SHH gene, while the cumulative release rate was calculated.

#### 2.7.7 *In vitro* proliferation experiment

The preparation of a solution of 3- (4,5-dimethylthiazol-2-yl) -2,5-diphenyl-tetrazolium bromide (MTT) was carried out as follows: 250 mg of MTT were dissolved in 50 mL of PBS in dark conditions and filtered with a 0.22 μm filter before storage at 4°C. Next, HA-F127 solution was prepared at 3.5% (w/v) with DMEM as stock solution. All the RMSCs were divided into 6 groups and cultivated with different concentrations of HA-F127 solution, including culture and diluted stock solution of HA-F127 (80%, 40%, 20%, and 10%), while each group was examined in quintuple. Then, the proliferation of different groups was calculated with MTT method. Briefly, 50 µl MTT solution were added into each well prior to 4 h incubation. Next, we discarded the supernatant, before addition of DMSO (150 µl). The samples were mixed, prior to measuring the absorbance value of each group at 490 nm with the enzyme-linked immunosorbent monitor.

#### 2.7.8 Dead/live assay

RMSCs were cultured in 3.5% HA-F127 and detected with Live/dead kit (Invitrogen, L3224) at different times, *viz.*, 24, 72, and 120 h according to the protocol. We washed the cells with PBS (pH 7.4) prior to observation and recording under florescent microscope. The number of living and dead cells was recorded, and the ratio of dead to live cells was calculated with ImageJ.

### 2.8 Repair of SCI in rats

#### 2.8.1 Establishment of rat SCI model

Rat SCI models were constructed as an existing study (Yu et al., 2021) (30). Briefly, anesthesia of male SD rats (200–250 g, 14–16 weeks old) was carried out *via* intraperitoneal injection of 10% (w/v) chloral hydrate (3.2 mL/kg, i.p.), and tying to an animal stereotaxic instrument, before hair trimming and disinfection of the skin. Later, laminectomy at the level of the T9−T11 vertebral column was performed to expose the spinal cord. Complete spinal cord transection at the T10 level was performed using a microscopic scissor after lifting the spinal cord with a self-made hook. The hook was passed through the gap to ensure that there were no residual fibers on the bottom and lateral sides of the canal. After the hemostasis, cells were added dropwise *in situ*. Finally, the skin and other tissues were sutured, while the gentamicin was injected for 4 days.

#### 2.8.2 Experiment design

Male SD rats (200–250 g) were randomized into four groups, namely sham operation (*n* = 10), model (*n* = 10), RMSC treated (*n* = 10) and SHH-RMSC treated groups (*n* = 40).

#### 2.8.3 Behavioral assessment

All experimented rats were scored through combination of inclined plate and BBB tests for 8 weeks. The scores were assigned into three grades of SCI repairing, as previously described by Basso et al. (1995), wherein rats that could move only its hind limbs minus support for its weight (0–8), rat that could support its weight without coordination (Lemons et al., 1999; Becker et al., 2000; Neuhuber et al., 2005; Chhabra and Arora, 2012; Reindl and Waters, 2019), and rat that could stabilize its trunk to coordinate its movement (Beresford et al., 1992; Lennon et al., 1995; Wakitani et al., 1995; Pittenger et al., 1999; Han et al., 2005; Rubio et al., 2005; Falanga et al., 2007; Lin et al., 2018). Through objective standards, we strictly evaluated the motor function of the hind limb. Later, the score were given by two independent researchers that had no idea about the experiment. Using the inclined plane test established by Rivlin and Tator (Rivlin and Tator, 1977), we placed the rats on an inclined board with their heads turned to the left with gradual increase in the angle from the horizontal position (0°). The score was calculated at the maximum angle at which the animal could stay on the board for 5 s without falling. All calculations were performed in triplicate.

#### 2.8.4 Immunohistochemical staining

Exploration of glial fibrillary acid protein (GFAP), growth-associated protein 43 (GAP43), myelin basic protein (MBP), and neurofilament 200 (NF200) expressions at protein level in the experimental group was accomplished by immunohistochemical staining. Paraffin-embedded tissue sections were firstly deparaffinized with 100% xylene, followed by rehydration using gradient ethanol and soaked in the boiled citric acid repair solution. Then, the sections were blocked with 5% BSA. The samples were stained with primary antibodies at 4°C overnight followed by incubation with secondary antibodies and SABC reagents. After that, staining of the slices were done with DAB, lightly counterstained with hematoxylin for 0.5–2 min before recording image with microscope.

#### 2.8.5 Histopathological study

After dewaxing the paraffin sections, the slices from all the groups were stained with hematoxylin eosin for 5 min before 10 min of washing with DD water. The slices were then treated with a 1% hydrochloric acid-ethanol solution for 30 before 30 s of washing with DD water. Subsequently, the slices were stained with 0.5% eosin for 1–3 min and washed with DD water for 30 s, before subsequent washing with 80 and 95% ethanol, absolute ethanol, and xylene for 1–3 min. After sealing with neutral gum, the slices were observed and imaged under microscope.

#### 2.8.6 Immunofluorescence

Paraffin sections were deparaffinized before we soaked (lasted 5–10 min) all the slices in 3% hydrogen peroxide, and thricely washed with PBS. Next, the slices were boiled in the citric acid repair solution and washed twice with PBS. And the sections were incubated in blocking solution of 5% BSA. The samples were stained with primary antibodies at 4°C overnight followed by incubation with fluorescently labeled secondary antibodies. Washing of the slices with PBS was performed before staining with DAPI for 15 min. Finally, antifluorescence quencher was dropwise added in the dark, prior to observation under a fluorescence microscope.

### 2.9 Statistical analysis

Presentation of data derived from the experiments was accomplished with mean and standard deviation (SD). Statistically, we evaluated differences within varied groups through ANOVA and least significant difference (LSD) test. Significance level was statistically accepted at *p* < 0.05. SPSS software (version 19.0, SPSS Inc., Chicago, United States) was used for calculations, while. Plotting of graphs was done with Origin Software® (Origin-Lab Corporation).

## 3 Results

### 3.1 Identification of RMSCs

**FIGURE 1:**
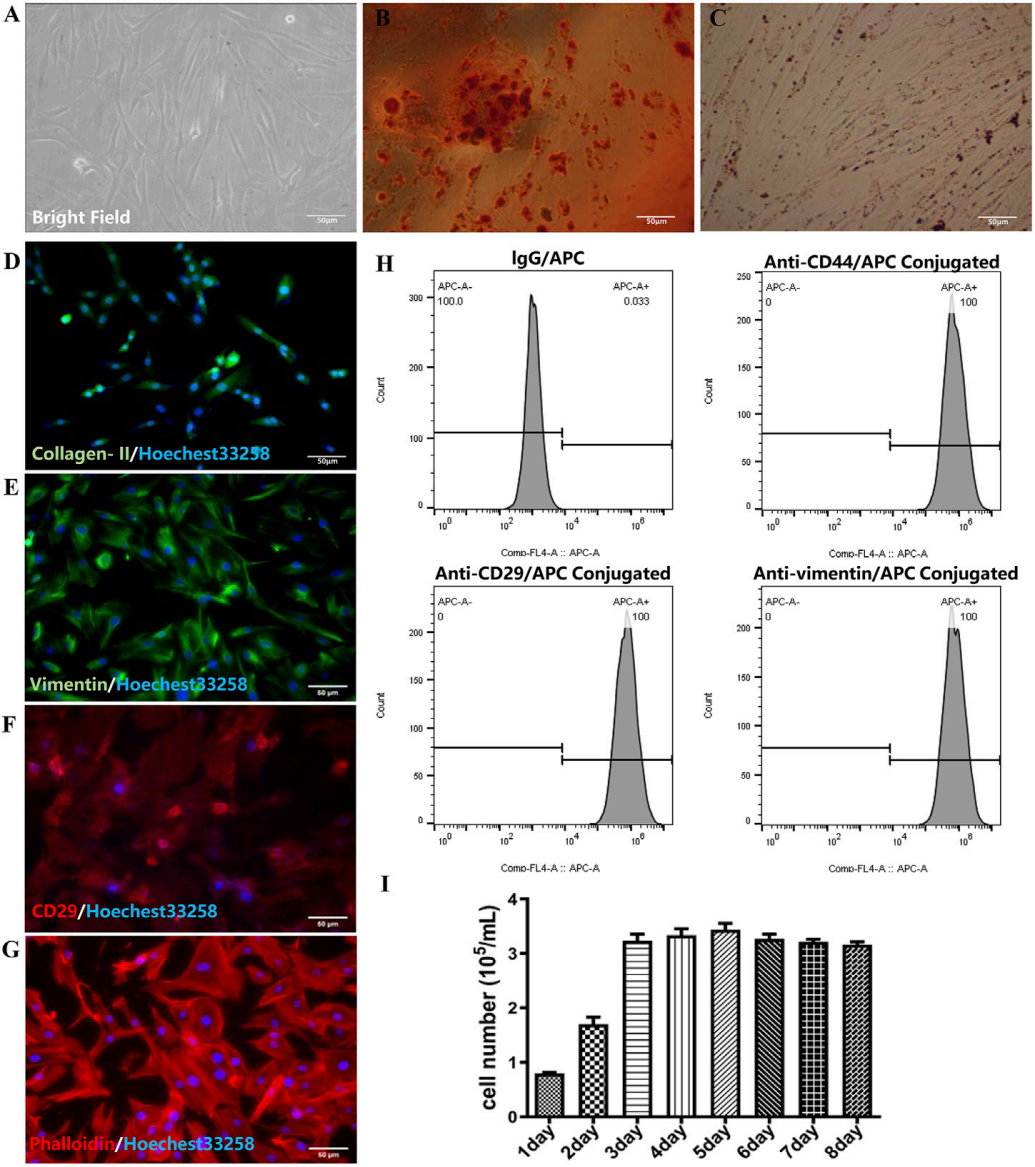
Identification of RMSC (scar = 50 μm). **(A)** Bright field image of RMSCs; **(B)** Adipogenic RMSCs were stained with Oil Red O; **(C)** Alizarin Red staining of osteogenic RMSCs; **(D)** collagen II immunofluorescence of chondrogenic RMSC; **(E)** Immunofluorescence staining of vimentin; **(F)** Immunofluorescence staining of CD29; **(G)** Immunofluorescence staining of phalloidin; **(H)** The flow-cytometry analysis of CD44, CD29, and vimentin; **(I)** The growth bar graph curve of RMSCs.

The fresh separated RMSCs were deposited on the beneath of culturing flask, which was a round shape and bright cytoplasm with good refraction. After 24 h, the RMSCs began to attach to the bottom, while the cytoplasm extended outward. The adherent cells appeared to be spindle, triangular, fan-shaped and round with fibroblasts like morphology after 48 h (as shown in Figure 1A). Agglomeration of lipid droplets could be seen in RMSCs after adipose induction, which proved adipogenesis of RMSCs (Figure 1B). In osteoblast inducted differentiation, calcium nodules could be detected in RMSCs which verified osteogenesis of RMSCs (Figure 1C). Then, the positive expression of collagen II displayed in Figure 1D illustrated the chondrogenesis of RMSCs. As shown in Figures 1E, F, H, RMSCs were highly positive for the surface markers CD29, CD44 and the intracellular markers vimentin. Meanwhile, the expression of phalloidin (Figure 1G) clearly shown the distribution of microfilament skeleton in RMSCs. The growth bar graph of MTT depicted that the cells proliferated rapidly with stable growth status (Figure 1I). In all, the results showed high purity, fast proliferation and stable biological characteristics of RMSCs.

### 3.2 Cationic liposomal preparation and characterization

**FIGURE 2:**
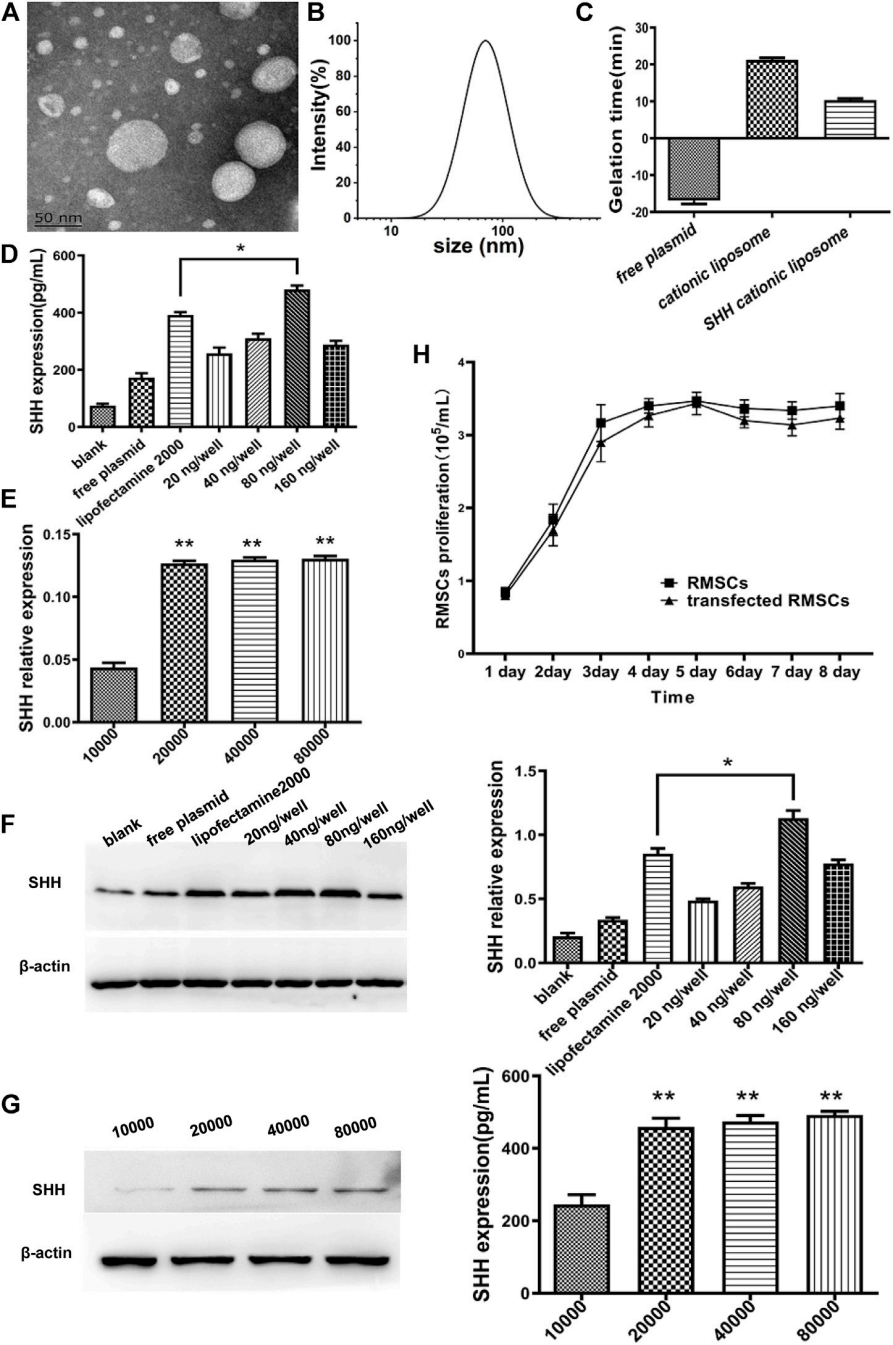
Characterization of cationic liposomes and screening for optimal SHH transfection conditions. **(A)** TEM of cationic liposomes; **(B)** The particle size distribution of cationic liposomes. **(C)** Zeta potential of plasmids, cationic liposomes, and SHH-cationic liposomes. **(D)** and **(F)** The SHH with different concentration of plasmids by ELISA and Western blotting (WB). The SHH expression of 80 ng per well was significantly higher than other groups and obtained better expression than lipofectamine 2000 (*, *p* < 0.05). **(E)** and **(G)** The SHH expression with different cell density by ELISA and WB. The best expression is in the condition of 80,000 cells (**, *p* < 0.01), and both 2000 cells and 4,000 cells were higher than 10,000 (**, *p* < 0.01). **(H)** The proliferation rate of RMSCs after transfection of the SHH gene from 1 to 8 days.

The average size of cationic liposomal particles was 85.76 ± 3.48 nm, whereas zeta potential was 15.76 ± 2.1 mV (Figures 2B,C). Also, TEM (Figure 2A) of cationic liposome displayed the same result as DLS with the liposomal particles being smaller (˂100 nm), which exhibited uniform globular shape. The SHH plasmid and cationic liposomal solution (without FBS) were mixed thoroughly at room temperature to prepare SHH-loaded liposomes, while its zeta potential was observed to be 10.37 ± 0.80 mV (Figure 2C).

### Screening for the best transfection ratio and studying of growth curve

Different concentrations of SHH plasmids (20, 40, 80, and 160 ng/well) and varied cell numbers (1 × 10^4^, 2 × 10^4^, 4 × 10^4^, 8 × 10^4^ cells per well) were examined with cationic liposomes to optimize the transfection condition. Through detection of SHH expression at protein level with ELISA and WB technique, we observed (Figures 2D–G) that compared with lipofactime 2000, the transfection efficiency of cationic liposomes was higher, whereas the optimal transfection concentration was reached when the plasmid concentration was 80 ng per well (*p* < 0.05) and 80,000 cells per well (*p* < 0.01). The growth curve (Figure 2H) was calculated through the MTT method, which displayed faster cell proliferation rate in the SHH transfected group compared to control group, thus indicating good growth status and low toxicity in the former group.

### 3.3 Preparation and characterization of thermo-sensitive gel

**FIGURE 3:**
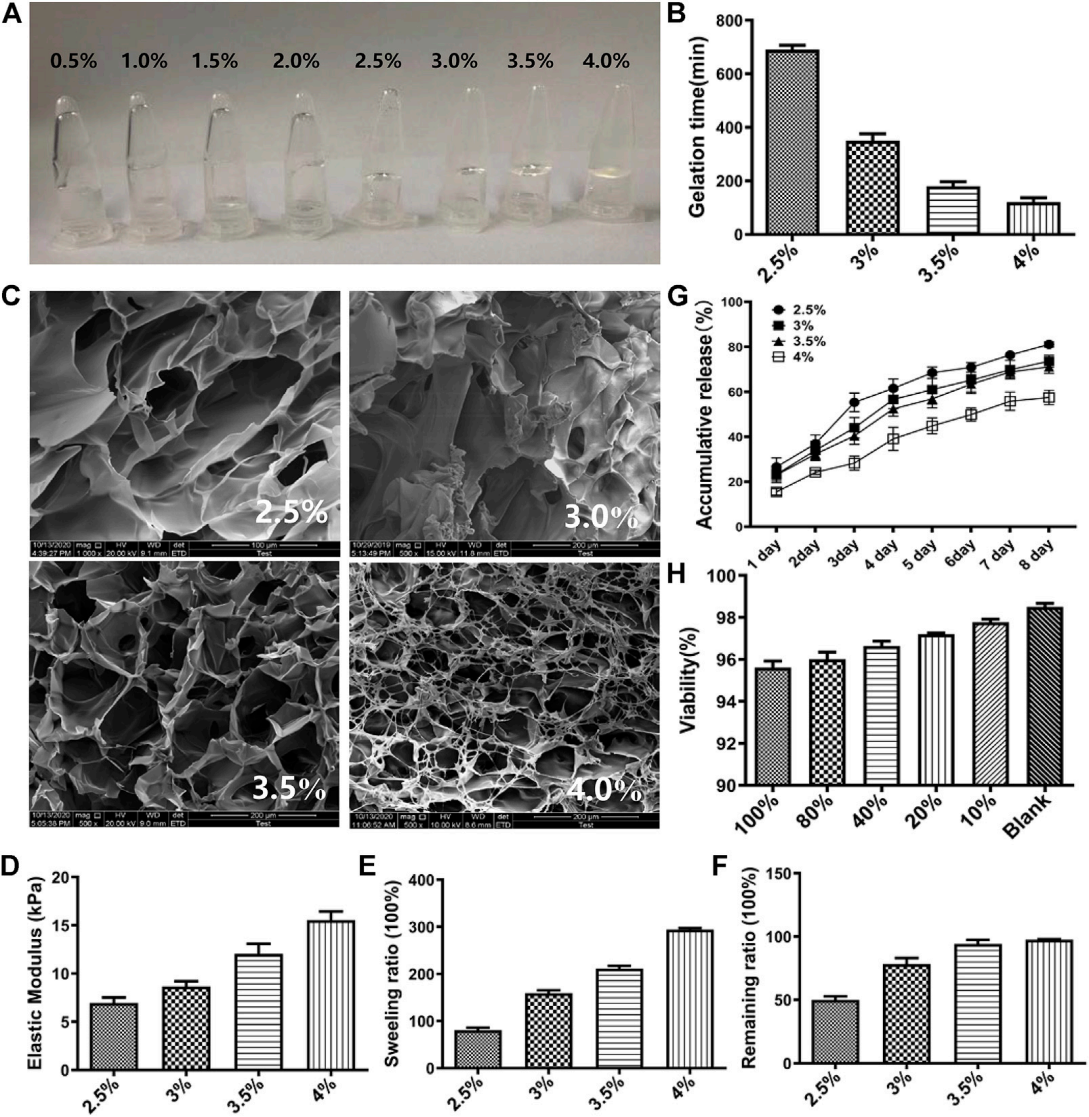
Characterization of HA-F127. **(A)** The appearance of 0.5%, 1.0%, 1.5%, 2.0%, 2.5%, 3.0%, 3.5%, 4.0% HA-F127. It was obvious that 0.5%, 1.0%, 1.5%, 2.0% HA-F127 did not solidified; **(B)** The gelation times of 2.5%, 3.0%, 3.5%, 4.0% HA-F127; **(C)** The TEM of 2.5%, 3.0%, 3.5%, 4.0% HA-F127; **(D)**, **(E)**, **(F)**, and **(G)** The elastic modulus, swelling ratio, remaining ratio and release of 2.5%, 3.0%, 3.5%, 4.0% HA-F127, respectively. **(H)** The RMSCs proliferation in under different concentration of HA-F127.

HA-F127 containing 0.5%, 1.0%, 1.5%, and 2.0% sodium hyaluronate did not remain coagulative. It can intuitively be found in Figure 3A that 0.5%, 1.0%, 1.5%, 2.0% sodium hyaluronate flowed to the bottom of the containers, while those composing of 2.5%, 3.0%, 3.5%, 4.0% gelatinized at the top of containers. The gelation times of 2.5%, 3.0%, 3.5%, 4.0% HA-F127 were 12, 6, 3, 2 min, respectively (Figure 3B). Therefore, the initial selected ratio of HA-F127 contained 2.5%, 3.0%, 3.5%, and 4.0%. The SEM results (Figure 3C) showed that the HA-F127 morphology of 3.0% and 3.5% had a more uniform porous structure. A more uniform porous structure provided more space for RMSC growth. The results of the mechanical compression test showed that the mechanical strength of the hydrogel was improved by the increased ratio of HA (Figure 3D). The results of the swelling degree test also demonstrated that the swelling degree of the hydrogel increased with increasing concentration of HA (Figure 3E). During combination of gel release finding (Figure 3F) and result of *in vitro* degradation tests (Figure 3G), we observed that when the ratio of HA was 4.0%, the cumulative release of the hydrogel scaffold was the smallest with the remaining *in vitro* being the highest. These could result in lower release of SHH and poor efficacy in SCI model after transplantation of SHH-transfected RMSCs combined with HA-F127. However, the higher cumulative release and the lower remaining ratio showed the poor stability of the HA-F127 structure, such as 2.5%, 3%. The cumulative release and degradation of 3.5% HA-F127 could provide a better microenvironment for the growth of RMSCs. Thus, 3.5% HA-F127 were selected to apply to the cell viability experiment. The stock solution was prepared by soaking 3.5% HA-F127 in DMEM solution. Then the RMSC viability was detected by MTT with different concentration of the stock solution, including 10%, 20%, 40%, 80%, and 100%. As shown in Figure 3H, the viability result showed that a low concentration of the stock solution demonstrated high cell proliferation, and that no stock solution (Blank) led to the highest viability. Though the viability was lower following the increasing stock concentration, the viability was also as high as 95.60% when the stock concentration was 100%, that showed that 3.5% HA-F127 was no significant suppression for RMSCs.

### 3.4 Live/dead assay

**FIGURE 4:**
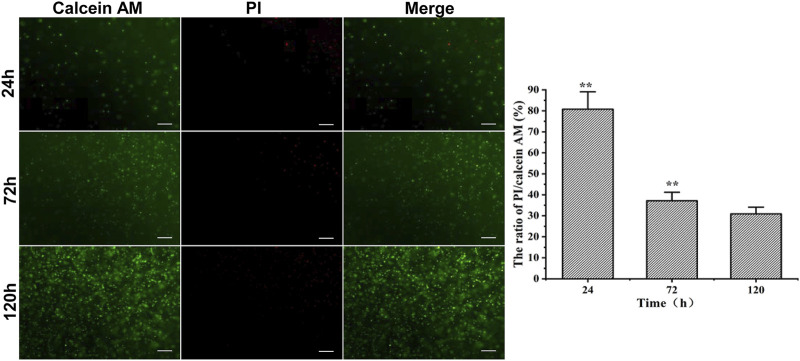
RMSC PI/Calinein AM assay in 3.5% HA-F127 at 24, 72, 120 h. 3.5% HA-F127 for 120 h showed that the ratio of the red fluorescence (dead)/green fluorescence (live) was significantly lower than 24 and 72 h (**, *p* < 0.01), indicating that the RMSCs obtained more better medium to survive and proliferate well in the hydrogel.

The RMSCs embedded in 3.5% HA-F127 were cultivated and stained for live/death assays at 24, 72, and 120 h, accordingly. As indicated in Figure 4, calcein AM (green) stained the live cells, while the PI (red) labeled the dead cells. It was found that the cell viability of 3.5% HA-F127 in 120 h was higher than 24 and 72 h (*p* < 0.01), while the viability in 72 h was higher than 24 h. Live/dead results indicated that the RMSCs survived and proliferated well in the 3.5% HA-F127.

### 3.5 Repair of SCI in rats

#### 3.5.1 The improvement of behavior in spinal cord injury

**FIGURE 5:**
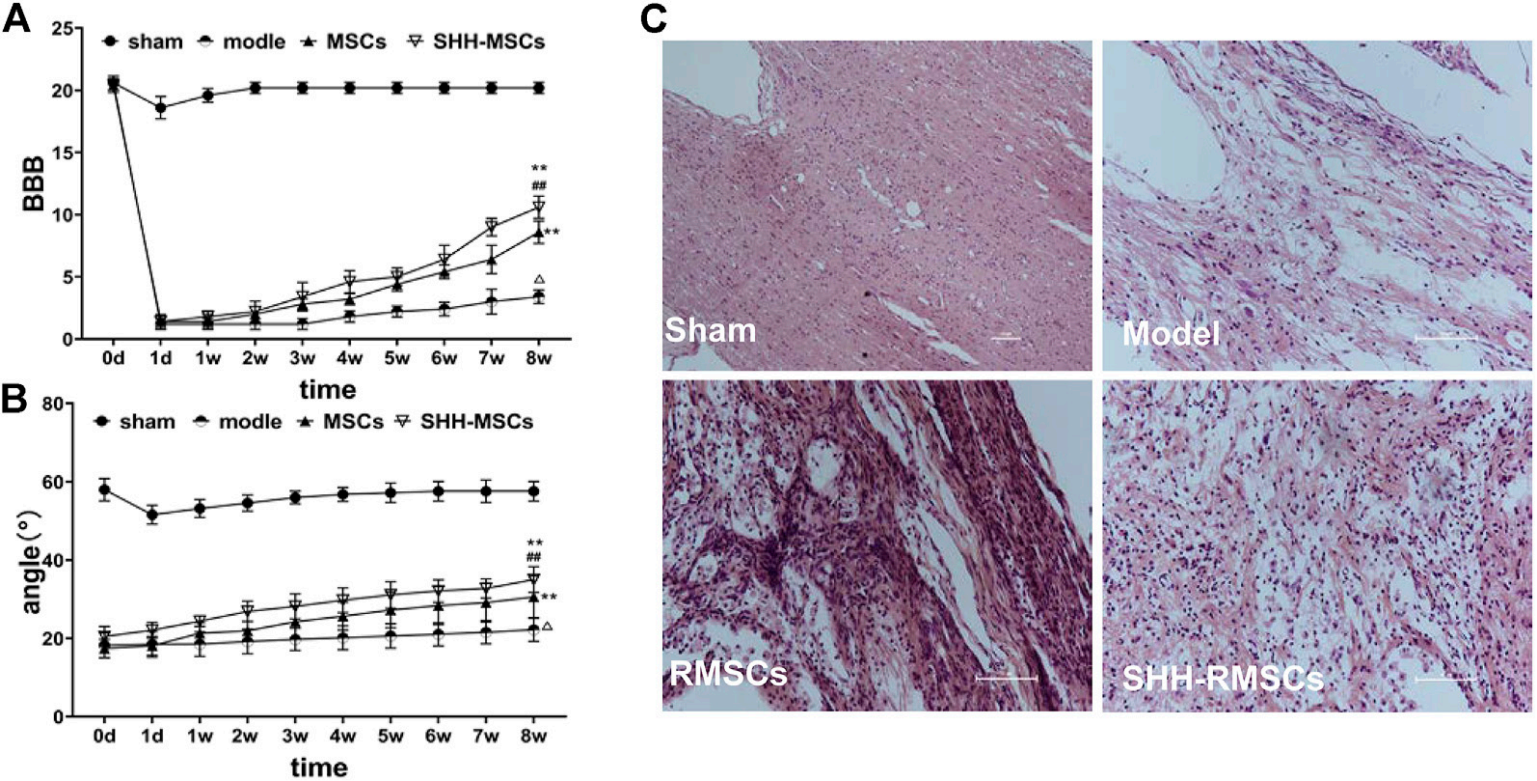
**(A)** and **(B)** The BBB scores and inclined plate angles of SCI for 8 weeks. The results of the SHH-RMSC group were significantly higher than those of the model, sham, and RMSC, and the difference was statistically significant (**, *p* < 0.01, compared with model; ^##^, *p* < 0.01, compared with MSCs; Δ, *p* < 0.01, compared with sham). **(C)** H&E staining of SCI. SHH-RMSCs group improved significantly, while the tissue arrangement tended to be normal, and the extracellular space was significantly reduced.

The BBB scores (Figure 5A) of all rats in each group were evaluated prior to surgery for SCI and were found to be in the range of 20–21. The BBB scores in the operated group dropped to 2 points after modeling, while the sham operated group only dropped to around 18–19 points coupled with rapid recovery. The BBB score of the model group remained at 2–3 in 1–8 weeks after surgery, indicating no significant recovery, while those of the RMSC and SHH-RMSC groups increased gradually. In the SHH-RMSCs group, the BBB score of the best recovered rats was 11, which increased substantially compared to other groups (*p* < 0.01).

As shown in inclined plate test (Figure 5B), angle of oblique plate test in normal rats in each group was 58–60°. The test angle of operated rats reduced to 17–20°, while the test angle of sham operated group remained basically unchanged. The test angle of the operated rats in the model group was basically unchanged around 18–20° in 1–8 weeks after surgery, while the rats in the RMSC and SHH-RMSC groups gradually increased. Furthermore, the maximum test angle of rats in the SHH-RMSC group was recovered to approximately 36°, which increased markedly compared to other groups (*p* < 0.01).

#### 3.5.2 Pathological observation of spinal cord tissue

As shown in Figure 5C, the SC tissue of the rats in the model group was still damaged after 8 weeks, i.e., the chromatin of neurons became sparse, some neurons appeared nuclear fragmented, cell bodies shrank, extracellular space increased and some nuclei dissociated. However, the SCI tissue of the RMSC and SHH-RMSC groups was improved. Among the different groups, we found that rats in the SHH-RMSCs group improved significantly as their tissue arrangement tended to be normal, while the extracellular space was reduced substantially.

#### 3.5.3 Immunohistochemistry and immunofluorescence

**FIGURE 6:**
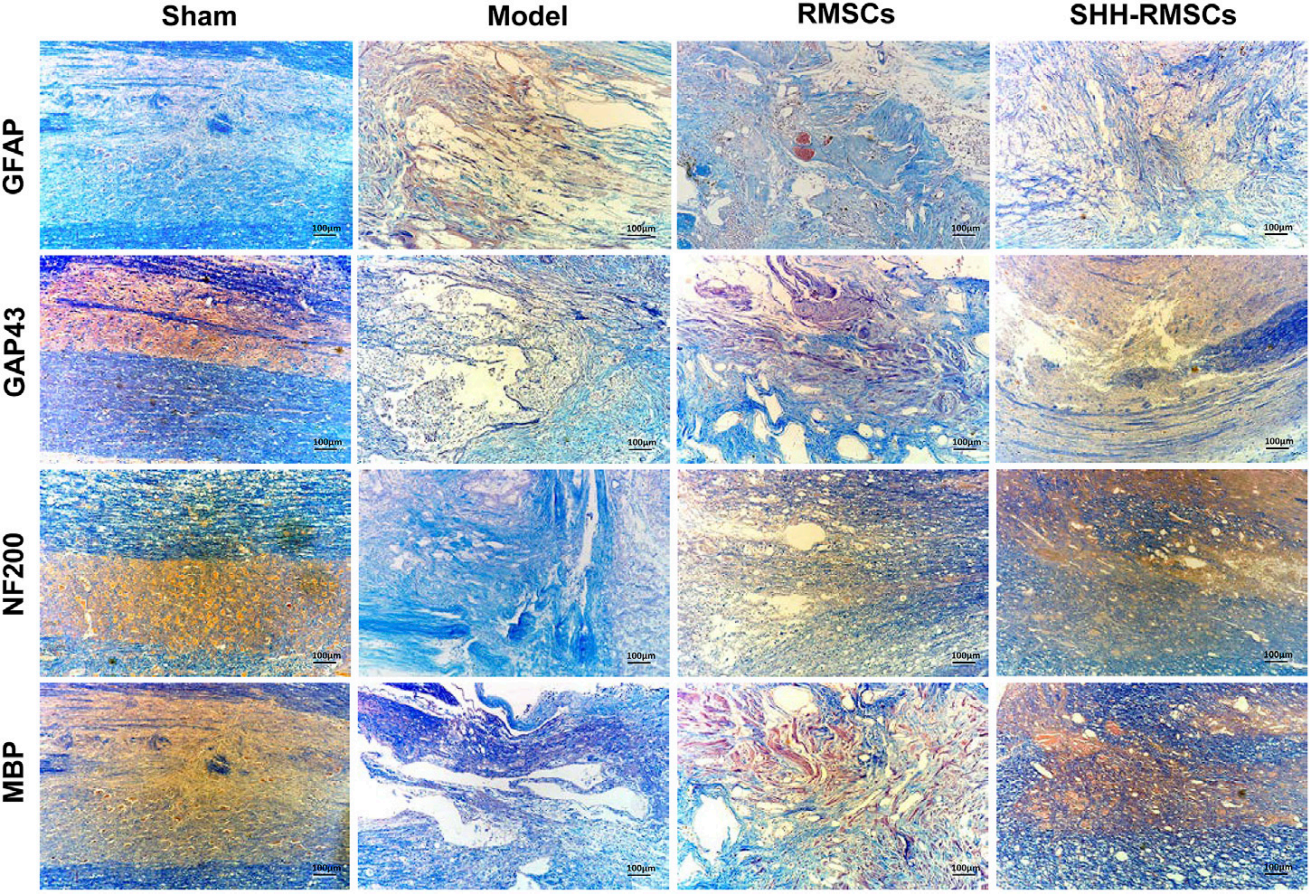
Immunohistochemistry of Sham, Model, RMSCs and SHH-RMSCs, including GAP43, NF200, MBP, and GFAP. SHH-RMSCs could significantly improve the expression of NF200, MBP, and GAP43, as well as inhibit GFAP expression.

**FIGURE 7:**
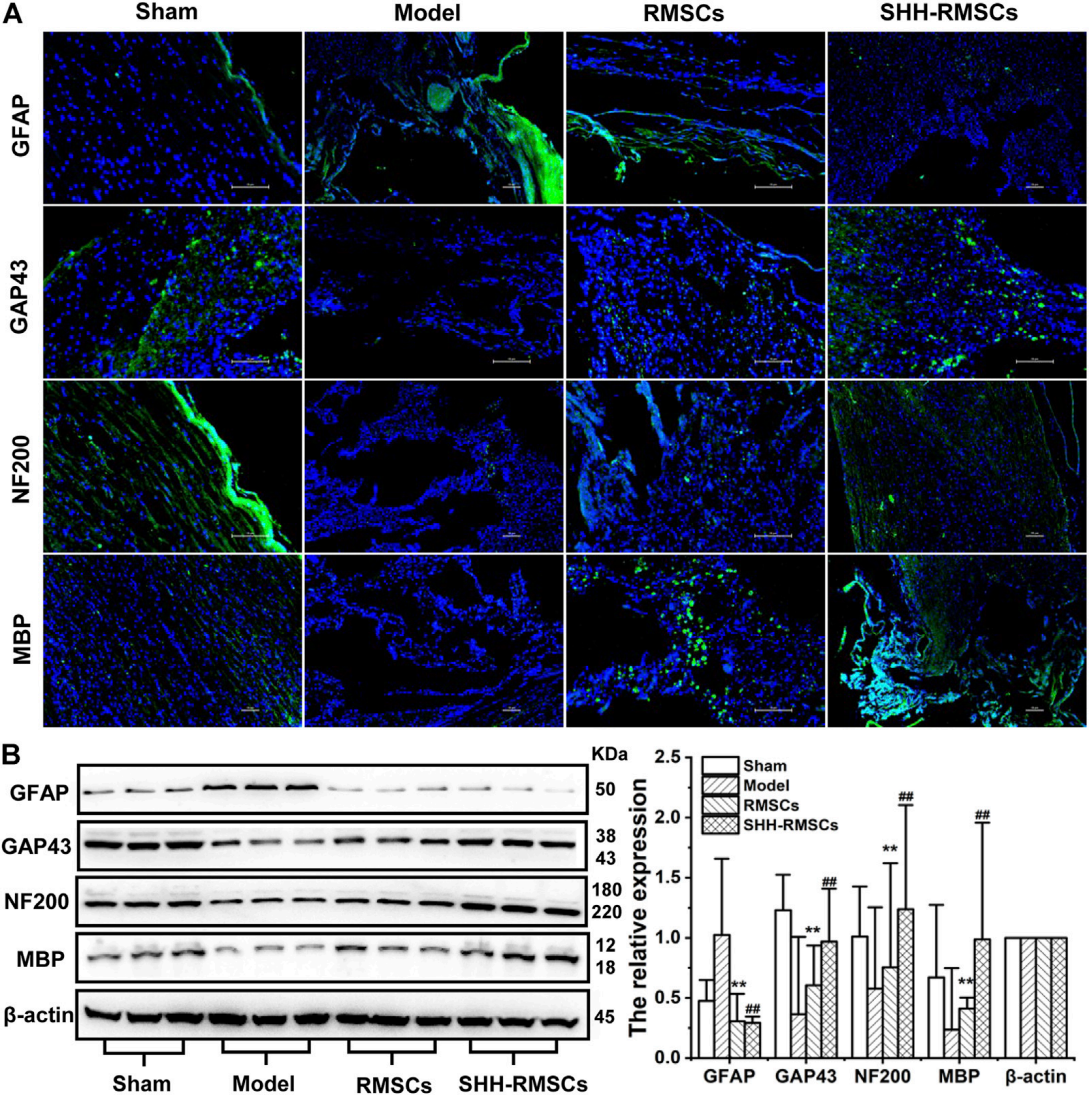
**(A)** The immunofluorescence of Sham, Model, RMSCs and SHH-RMSCs, including GAP43, NF200, MBP, and GFAP. SHH-RMSCs could significantly improve the expression of NF200, MBP, and GAP43, but inhibit the expression of GFAP. **(B)** The protein expression of GAP43, NF200, MBP, and GFAP in the Sham, Model, RMSCs, and SHH-RMSC groups. (**,^##^, *p* < 0.01, compared with model).

Levels of GAP43, NF200, MBP, and GFAP in the four groups were detected by immunofluorescence and immunohistochemistry (Figure 6 and Figure 7A). GAP43, NF200, and MBP expression levels in the model group were the lowest, suggesting that the structure of the SC tissue was significantly damaged. In contrast, we observed a substantial increase in expression of GAP43, NF200 and MBP in the RMSCs and SHH-RMSCs groups, while the SHH-RMSCs group exhibited a significant improvement in the SCI tissue. Meanwhile, we observed highest GFAP expression in the model group, whereas a decreased expression of these genes was found in the RMSCs and SHH-RMSCs groups. Also, the GFAP expression in SHH-RMSCs group was the lowest, thereby verifying that the SC tissue of rats in this group improved markedly.

#### 3.5.4 The protein expression of GAP43, NF200, MBP, and GFAP

GAP43, NF200, MBP, and GFAP protein levels in the four groups were detected by Western blotting (Figure 7B). In the RMSCs and SHH-RMSCs groups, we observed a significant up-regulation in expression of GAP43, NF200 and MBP (*p* < 0.01), and a down-regulated expression of GFAP (*p* < 0.01).

## 4 Discussion

**FIGURE 8:**
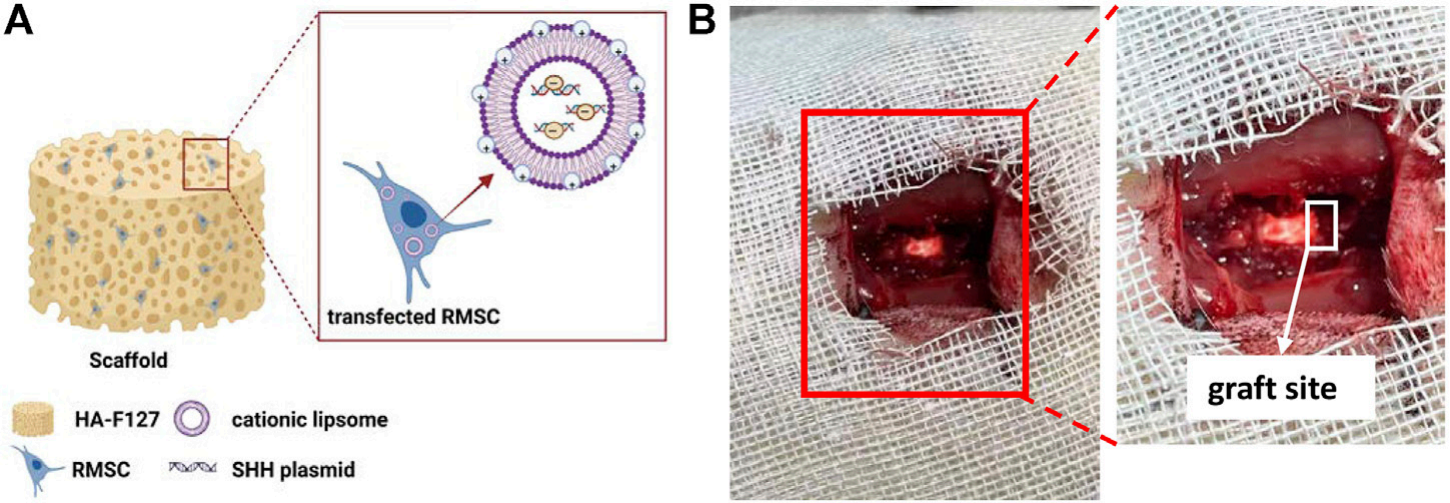
The schematic diagrams. **(A)** The composition of graft; **(B)** The graft site in spinal cord injury models.

In recent years, the study of RMSCs has been of great interest in the fields of regenerative medicine and animal biotechnology alike. In our study, we combined the thermo-sensitive hydrogel (3.5% HA-F127) with SHH expressed RMSCs as Figure 8A, then transplanted the mixture (SHH-RMSCs-loaded HA-F127) into the injured section in SCI rats as Figure 8B. This treatment could significantly improve behaviors and enhanced nerve regeneration in SCI rats.

Similar to Jaromír Vašíček’s work (Vašíček et al., 2020), we also successfully isolated RMSCs from rats, these cells have presented positive expression of CD29, CD44 and vimentin (Figure 1). Then we prepared cationic liposomes as carriers to transfer the SHH into RMSCs, amidst the effect of transfection being higher than lipofectamine 2000 (Figure 2D). As a common non-viral vector, cationic liposomes have a lot of advantages, such as safety, simple preparation and high transfection efficiency. Also, a stabilized complex was formed when cationic liposomes interacted with negative charge of the target gene, which can transfer various substances into different types of cells (Zdanov et al., 2000). Several studies have found that cationic liposomes to be a carrier, exhibited much better transfection efficiency in the HEK293 cell lines (Wu et al., 2022), a mouse neuroblastoma cell line (Yang et al., 2019), HeLa cells (Paecharoenchai et al., 2012) and dendritic cells (Markov et al., 2012). Interestingly, we chose RMSCs that transfected SHH DNA *via* cationic liposomes and expect RMSCs to improve neurology recovery in our study. The SHH plays a crucial role in nervous development by stimulating production of neuron and oligodendrocyte from ventral cord progenitor cells, enhancement of synaptic plasticity, inhibition of astrocyte formation and guidance of axon guidance growth (Charron et al., 2003; Gulino and Gulisano, 2013; Ogura et al., 2018). Here, SHH-loaded cationic liposomes maintained a higher transfection rate under 80 ng/well and 8 × 104 cells/well conditions compared to lipofectamine 2000. Under this condition, it is possible that the liposome may have borne an overall positive charge, thereby enabling it to fully bind with negatively charged components of cell membrane to obtain higher transfection efficiency (Felgner et al., 1997; Dodds et al., 1998).

Meanwhile, we prepared HA-F127, a thermo-sensitive hydrogel scaffold, which created an environment for the cells to attach because of its three-dimensional network structure. When the HA concentration was 3.5%, HA-F127 could obtain a porous structure with a uniform pore size in a shorter time. The cumulative release, degradation and mechanical compression were also better than other ratios. Importantly, RMSCs in HA-F127 could live normally for a long time according to the results of the dead/living assay. As a kind of natural macromolecular glycosaminoglycan with good biocompatibility and biodegradability, HA is easily decomposed by HA enzyme *in vivo* (How et al., 2020). Pluronic F127 is a temperature-sensitive non-ionic triblock copolymer composed of a hydrophobic polyoxypropylene (PPO) chain in the middle and a hydrophilic polyoxyethylene at both ends (PEO) (Charron et al., 2003). Combining F127 with HA did not only protect effectively HA from degradation due to hydrophilic PEO segment of F-127, but also encapsulate hydrophobic drugs as a result of hydrophobic PPO segment of F-127 (Bae et al., 2006; Bae et al., 2007; Zhang et al., 2010). At present, SCI treatment is still complex and challenging. Conventional hydrogels including chitosan, gelation, poloxamer 407 and hydroxyethyl methacrylate could not provide a good environment, which leading to inhibition of nerve repair (Zhao et al., 2016). We suspected that the combination of SHH-transfected cationic liposomes with HA-F127 may promote more effective repair in the SCI.

The SD rat SCI model was then established, while grafts (SHH-transfected liposomes and HA-F127, as Figure 8A) were applied at the site of the injury as in Figure 8B. From *in vivo* study, the behavior of rats in the treated group improved significantly by improvement of BBB test and inclined plate test. Also, these results need to be interpreted with more methods, such as the open-field test and the record of fore and hind paws. It was obvious that GAP43, NF200, and MBP were significantly expressed at the site of injury, whereas GFAP was inhibited in the SHH-RMSCs group as indicated in the results of immunohistochemistry and immunofluorescence. GAP43 and NF200 are specific proteins in the nervous system, which are closely related to nerve growth (Al-Chalabi and Miller, 2003; Shen et al., 2008; Harauz and Boggs, 2013). GFAP is the marker of astrocytes that proliferate rapidly during nerve injury. Astrocytes can induce and support nerve fiber regeneration of nerve fiber in the early stage of injury, and they have a negative effect on SCI repair in the later stage of damage, due to mechanical obstruction of the glial scars and inhibition of secreted chemicals (Shih et al., 2003). The inhibition of GFAP coupled with upgrade of GAP43, NF200 and MBP indicated that the injured site received a better restoration. In addition, we cannot exclude a potential effect of transplanted RMSCs, the fate of transplanted RMSCs in the host tissue should be tracked. From pathological observation, the SHH-RMSCs improved significantly and tended to be normal, while the extracellular space was substantially reduced. SCI results in a series of primary and secondary injuries, including inhibition of neuronal function due to loss of neural tissues, suppression of nerve cell growth due to glial scar formation, and restriction of SCI recovery due to up-regulation of inhibitory molecules (Block et al., 2007). It was obvious that the potential of SHH-RMSCs-loaded HA-F127 on nerve regeneration for SCI could be realized under *in situ* administration.

## 5 Conclusion

We have prepared cationic liposomes as nonviral gene carriers to transfect SHH into RMSCs. Meanwhile, HA-F127, as a suitable temperature sensitive hydrogel, is safe and suitable for cell growth due to its high biocompatibility. We embedded SHH transfected RMSCs in HA-F127 (3.5%) gel for the treatment of SCI. Assays showed that the SHH treated RMSC group had a better effect on SCI repair, including improvement of BBB and angle scores, higher expression of GAP43, NF200, and MBP and reduced expression of GFAP. These findings demonstrated that cationic liposomes could provide a safe and efficient platform for gene delivery, while RMSC transfected with SHH could promote SCI repair and enhance nerve regeneration in SCI.

## Data Availability

The original contributions presented in the study are included in the article/Supplementary material, further inquiries can be directed to the corresponding authors.

## Supplementary material

SUPPLEMENTARY FIGURE S1 5(6)-Carboxyfluorescein diacetate N-succinimidyl ester (CFSE) staining of RMSCs in spinal cord injury models. 5μmol/L CFSE was combined with RMSCs, and transplanted in to injury site. And green represented the CFSE marked RMSCs and blue was the cell nucleus.

## References

[B1] Al-Chalabi A. Miller C. C. (2003). Neurofilaments and neurological disease. Bioessays 25 (4), 346–355. 10.1002/bies.10251 12655642

[B2] Bae K. H. Choi S. H. Park S. Y. Lee Y. Park T. G. (2006). Thermosensitive pluronic micelles stabilized by shell cross-linking with gold nanoparticles. Langmuir 22 (14), 6380–6384. 10.1021/la0606704 16800702

[B3] Bae K. H. Lee Y. Park T. G. (2007). Oil-encapsulating PEO-PPO-PEO/PEG shell cross-linked nanocapsules for target-specific delivery of paclitaxel. Biomacromolecules 8 (2), 650–656. 10.1021/bm0608939 17291088

[B4] Bao R. Wang Q.-L. Li R. Adu-Frimpong M. Toreniyazov E. Ji H. (2020). Improved oral bioavailability and target delivery of 6-shogaol via vitamin E TPGS-modified liposomes: Preparation, *in-vitro* and *in-vivo* characterizations. J. Drug Deliv. Sci. Technol. 59, 101842. 10.1016/j.jddst.2020.101842

[B5] Basso D. M. Beattie M. S. Bresnahan J. C. (1995). A sensitive and reliable locomotor rating scale for open field testing in rats. J. Neurotrauma 12 (1), 1–21. 10.1089/neu.1995.12.1 7783230

[B6] Becker T. Anliker B. Becker C. G. Taylor J. Schachner M. Meyer R. L. (2000). Tenascin-R inhibits regrowth of optic fibers *in vitro* and persists in the optic nerve of mice after injury. Glia 29 (4), 330–346. 10.1002/(sici)1098-1136(20000215)29:4<330::aid-glia4>3.0.co;2-l 10652443

[B7] Beresford J. N. Bennett J. H. Devlin C. Leboy P. S. Owen M. E. (1992). Evidence for an inverse relationship between the differentiation of adipocytic and osteogenic cells in rat marrow stromal cell cultures. J. Cell Sci. 102 (2), 341–351. 10.1242/jcs.102.2.341 1400636

[B8] Block M. L. Zecca L. Hong J. S. (2007). Microglia-mediated neurotoxicity: Uncovering the molecular mechanisms. Nat. Rev. Neurosci. 8 (1), 57–69. 10.1038/nrn2038 17180163

[B9] Charron F. Stein E. Jeong J. McMahon A. P. Tessier-Lavigne M. (2003). The morphogen sonic hedgehog is an axonal chemoattractant that collaborates with netrin-1 in midline axon guidance. Cell 113 (1), 11–23. 10.1016/s0092-8674(03)00199-5 12679031

[B10] Chhabra H. S. Arora M. (2012). Demographic profile of traumatic spinal cord injuries admitted at Indian spinal injuries centre with special emphasis on mode of injury: A retrospective study. Spinal Cord. 50 (10), 745–754. 10.1038/sc.2012.45 22584285

[B11] Dodds E. Dunckley M. G. Naujoks K. Michaelis U. Dickson G. (1998). Lipofection of cultured mouse muscle cells: A direct comparison of lipofectamine and DOSPER. Gene Ther. 5 (4), 542–551. 10.1038/sj.gt.3300604 9614580

[B12] Falanga V. Iwamoto S. Chartier M. Yufit T. Butmarc J. Kouttab N. (2007). Autologous bone marrow-derived cultured mesenchymal stem cells delivered in a fibrin spray accelerate healing in murine and human cutaneous wounds. Tissue Eng. 13 (6), 1299–1312. 10.1089/ten.2006.0278 17518741

[B13] Felgner P. L. Barenholz Y. Behr J. P. Cheng S. H. Cullis P. Huang L. (1997). Nomenclature for synthetic gene delivery systems. Hum. Gene Ther. 8 (5), 511–512. 10.1089/hum.1997.8.5-511 9095402

[B14] Gulino R. Gulisano M. (2013). Noggin and Sonic hedgehog are involved in compensatory changes within the motoneuron-depleted mouse spinal cord. J. Neurol. Sci. 332 (1-2), 102–109. 10.1016/j.jns.2013.06.029 23859181

[B15] Hagen E. M. (2015). Acute complications of spinal cord injuries. World J. Orthop. 6 (1), 17–23. 10.5312/wjo.v6.i1.17 25621207 PMC4303786

[B16] Han S. K. Yoon T. H. Lee D. G. Lee M. A. Kim W. K. (2005). Potential of human bone marrow stromal cells to accelerate wound healing *in vitro* . Ann. Plast. Surg. 55 (4), 414–419. 10.1097/01.sap.0000178809.01289.10 16186710

[B17] Harauz G. Boggs J. M. (2013). Myelin management by the 18.5-kDa and 21.5-kDa classic myelin basic protein isoforms. J. Neurochem. 125 (3), 334–361. 10.1111/jnc.12195 23398367 PMC3700880

[B18] How K. N. Yap W. H. Lim C. L. H. Goh B. H. Lai Z. W. (2020). Hyaluronic acid-mediated drug delivery system targeting for inflammatory skin diseases: A mini review. Front. Pharmacol. 11, 1105. 10.3389/fphar.2020.01105 32848737 PMC7397973

[B19] Kerstetter A. E. Miller R. H. (2012). Isolation and culture of spinal cord astrocytes. Methods Mol. Biol. 814, 93–104. 10.1007/978-1-61779-452-0_7 22144302 PMC3568947

[B20] Lemons M. L. Howland D. R. Anderson D. K. (1999). Chondroitin sulfate proteoglycan immunoreactivity increases following spinal cord injury and transplantation. Exp. Neurol. 160 (1), 51–65. 10.1006/exnr.1999.7184 10630190

[B21] Lennon D. P. Haynesworth S. E. Young R. G. Dennis J. E. Caplan A. I. (1995). A chemically defined medium supports *in vitro* proliferation and maintains the osteochondral potential of rat marrow-derived mesenchymal stem cells. Exp. Cell Res. 219 (1), 211–222. 10.1006/excr.1995.1221 7628536

[B22] Lin G. L. Wang H. Dai J. Li X. Guan M. Ding Q. (2018). Upregulation of UBAP2L in bone marrow mesenchymal stem cells promotes functional recovery in rats with spinal cord injury. Curr. Med. Sci. 38 (6), 1081–1089. 10.1007/s11596-018-1987-x 30536073

[B23] Liu L. Feng X. Pei Y. Wang J. Ding J. Chen L. (2018). α-Cyclodextrin concentration-controlled thermo-sensitive supramolecular hydrogels. Mater. Sci. Eng. C 82, 25–28. 10.1016/j.msec.2017.08.045 29025655

[B24] Markov O. O. Mironova N. L. Maslov M. A. Petukhov I. A. Morozova N. G. Vlassov V. V. (2012). Novel cationic liposomes provide highly efficient delivery of DNA and RNA into dendritic cell progenitors and their immature offsets. J. Control. Release 160 (2), 200–210. 10.1016/j.jconrel.2011.11.034 22155599

[B25] Neuhuber B. Timothy Himes B. Shumsky J. S. Gallo G. Fischer I. (2005). Axon growth and recovery of function supported by human bone marrow stromal cells in the injured spinal cord exhibit donor variations. Brain Res. 1035 (1), 73–85. 10.1016/j.brainres.2004.11.055 15713279

[B26] Ogura T. Sakaguchi H. Miyamoto S. Takahashi J. (2018). Three-dimensional induction of dorsal, intermediate and ventral spinal cord tissues from human pluripotent stem cells. Development 145 (16). 10.1242/dev.162214 PMC6124545 30061169

[B27] Paecharoenchai O. Niyomtham N. Apirakaramwong A. Ngawhirunpat T. Rojanarata T. Yingyongnarongkul B. E. (2012). Structure relationship of cationic lipids on gene transfection mediated by cationic liposomes. AAPS PharmSciTech 13 (4), 1302–1308. 10.1208/s12249-012-9857-5 23007192 PMC3513429

[B28] Pittenger M. F. Mackay A. M. Beck S. C. Jaiswal R. K. Douglas R. Mosca J. D. (1999). Multilineage potential of adult human mesenchymal stem cells. Science 284 (5411), 143–147. 10.1126/science.284.5411.143 10102814

[B29] Reindl M. Waters P. (2019). Myelin oligodendrocyte glycoprotein antibodies in neurological disease. Nat. Rev. Neurol. 15 (2), 89–102. 10.1038/s41582-018-0112-x 30559466

[B30] Rivlin A. S. Tator C. H. (1977). Objective clinical assessment of motor function after experimental spinal cord injury in the rat. J. Neurosurg. 47 (4), 577–581. 10.3171/jns.1977.47.4.0577 903810

[B31] Rubio D. Garcia-Castro J. Martín M. C. de la Fuente R. Cigudosa J. C. Lloyd A. C. (2005). Spontaneous human adult stem cell transformation. Cancer Res. 65 (8), 3035–3039. 10.1158/0008-5472.Can-04-4194 15833829

[B32] Schnell L. Schwab M. E. (1990). Axonal regeneration in the rat spinal cord produced by an antibody against myelin-associated neurite growth inhibitors. Nature 343 (6255), 269–272. 10.1038/343269a0 2300171

[B33] Schwartz M. (2000). Autoimmune involvement in CNS trauma is beneficial if well controlled. Prog. Brain Res. 128, 259–263. 10.1016/s0079-6123(00)28023-0 11105685

[B34] Shen Y. Mishra R. Mani S. Meiri K. F. (2008). Both cell-autonomous and cell non-autonomous functions of GAP-43 are required for normal patterning of the cerebellum *in vivo* . Cerebellum 7 (3), 451–466. 10.1007/s12311-008-0049-5 18777197 PMC4164963

[B35] Shih A. Y. Johnson D. A. Wong G. Kraft A. D. Jiang L. Erb H. (2003). Coordinate regulation of glutathione biosynthesis and release by Nrf2-expressing glia potently protects neurons from oxidative stress. J. Neurosci. 23 (8), 3394–3406. 10.1523/jneurosci.23-08-03394.2003 12716947 PMC6742304

[B36] Taweel W. A. Seyam R. (2015). Neurogenic bladder in spinal cord injury patients. Res. Rep. Urol. 7, 85–99. 10.2147/rru.S29644 26090342 PMC4467746

[B37] Vašíček J. Kováč M. Baláži A. Kulíková B. Tomková M. Olexiková L. (2020). Combined approach for characterization and quality assessment of rabbit bone marrow-derived mesenchymal stem cells intended for gene banking. N. Biotechnol. 54, 1–12. 10.1016/j.nbt.2019.08.001 31400479

[B38] Villanova Junior J. A. Fracaro L. Rebelatto C. L. K. da Silva A. J. Barchiki F. Senegaglia A. C. (2020). Recovery of motricity and micturition after transplantation of mesenchymal stem cells in rats subjected to spinal cord injury. Neurosci. Lett. 734, 135134. 10.1016/j.neulet.2020.135134 32531527

[B39] Wakitani S. Saito T. Caplan A. I. (1995). Myogenic cells derived from rat bone marrow mesenchymal stem cells exposed to 5-azacytidine. Muscle Nerve 18 (12), 1417–1426. 10.1002/mus.880181212 7477065

[B40] Widenfalk J. Lundströmer K. Jubran M. Brene S. Olson L. (2001). Neurotrophic factors and receptors in the immature and adult spinal cord after mechanical injury or kainic acid. J. Neurosci. 21 (10), 3457–3475. 10.1523/jneurosci.21-10-03457.2001 11331375 PMC6762497

[B41] Wills A. Ninness B. (2012). Generalised Hammerstein-Wiener system estimation and a benchmark application. Control Eng. Pract. 20 (11), 1097–1108. 10.1016/j.conengprac.2012.03.011

[B42] Wu S. Liu M. Hu X. He C. Zhao C. Xiang S. (2022). Evaluation of pentaerythritol-based and trimethylolpropane-based cationic lipidic materials for gene delivery. Bioorg. Med. Chem. Lett. 62, 128635. 10.1016/j.bmcl.2022.128635 35202809

[B43] Yang B. Mao J. Jiang S. Wei J. Li Y. Gao B. (2019). Cholesterol depletion induced by RNA interference targeting DHCR24 protects cells from liposome-induced cytotoxicity. Prep. Biochem. Biotechnol. 49 (5), 453–458. 10.1080/10826068.2019.1591979 30896287

[B44] Yang W. Wang Z. Zhang J. Yang K. Lu C. Cui X. (2020). Fibrin scaffolds embedded with sonic hedgehog/chitosan microspheres for recovery of spinal cord injury in rats. Mat. express 10 (3), 437–445. 10.1166/mex.2020.1654

[B45] Yu Q. Liao M. Sun C. Zhang Q. Deng W. Cao X. (2021). LBO-EMSC hydrogel serves a dual function in spinal cord injury restoration via the PI3K-Akt-mTOR pathway. ACS Appl. Mat. Interfaces 13 (41), 48365–48377. 10.1021/acsami.1c12013 34633177

[B46] Yue Y. Zhao J. Li X. Zhang L. Su Y. Fan H. (2020). Involvement of Shh/Gli1 signaling in the permeability of blood-spinal cord barrier and locomotion recovery after spinal cord contusion. Neurosci. Lett. 728, 134947. 10.1016/j.neulet.2020.134947 32276104

[B47] Zdanov R. I. Semenova N. V. Archakov A. I. (2000). Realities and hopes of gene therapy. Vopr. Med. Khim. 46 (3), 197–206. 11033881

[B48] Zhang W. Gilstrap K. Wu L. Moss M. A. Wang Q. Lu X. (2010). Synthesis and characterization of thermally responsive Pluronic F127-chitosan nanocapsules for controlled release and intracellular delivery of small molecules. ACS Nano 4 (11), 6747–6759. 10.1021/nn101617n 21038924

[B49] Zhang X. Li L. Ouyang J. Zhang L. Xue J. Zhang H. (2021). Electroactive electrospun nanofibers for tissue engineering. Nano Today 39, 101196. 10.1016/j.nantod.2021.101196

[B50] Zhao Y. Z. Jiang X. Xiao J. Lin Q. Yu W. Z. Tian F. R. (2016). Using NGF heparin-poloxamer thermosensitive hydrogels to enhance the nerve regeneration for spinal cord injury. Acta Biomater. 29, 71–80. 10.1016/j.actbio.2015.10.014 26472614 PMC7517710

[B51] Zhong Z. R. Zhang Z. R. Deng Y. Liu J. Song Q. G. Liu J. (2007). Preparation of a TK/GCV administration system mediated by transferrin modified pro-cationic liposomes. Pharmazie 62 (7), 522–527. 17718194

[B52] Zhou L. Fan L. Yi X. Zhou Z. Liu C. Fu R. (2018). Soft conducting polymer hydrogels cross-linked and doped by tannic acid for spinal cord injury repair. ACS Nano 12 (11), 10957–10967. 10.1021/acsnano.8b04609 30285411

