# Supplementary figures and images for: Thermo-sensitive hydrogel combined with SHH expressed RMSCs for rat spinal cord regeneration
Source: Front Bioeng Biotechnol. 2022 Oct 21;10:1001396. doi: 10.3389/fbioe.2022.1001396 (PMC9634076; doi:10.3389/fbioe.2022.1001396)

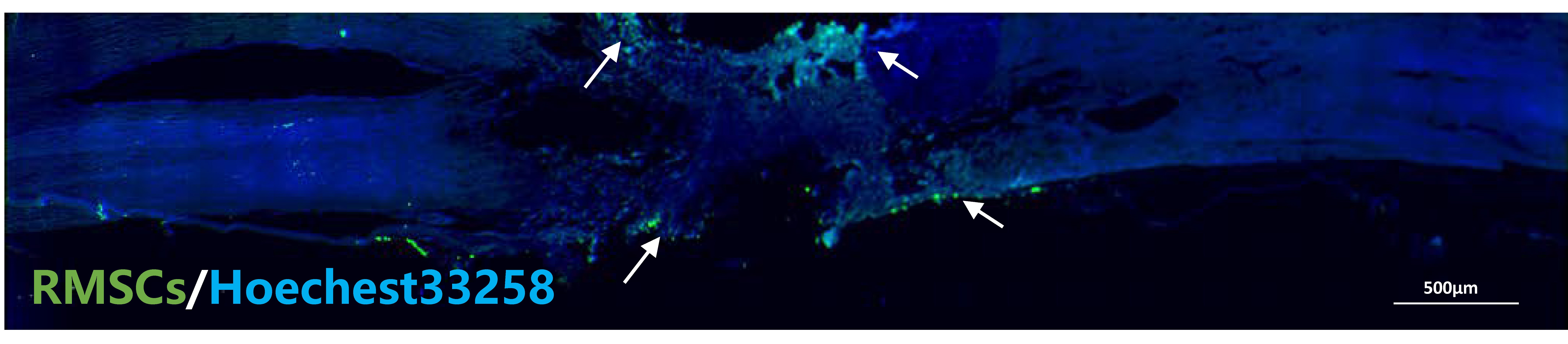

Supplement: Supplementary file 1 [file Image1.JPEG]
